# Artificial intelligence in breast ultrasound: a systematic review of research advances

**DOI:** 10.3389/fonc.2025.1619364

**Published:** 2025-09-30

**Authors:** Jiawei Liu, Linping Pian, Jie Chen, Jingjing Zhao, Yameng Liu, Fanbo Meng, Cheng Zeng

**Affiliations:** 1 Henan University of Traditional Chinese Medicine, Zhengzhou, China; 2 The First Affiliated Hospital of Henan University of Traditional Chinese Medicine, Zhengzhou, China; 3 The Third Affiliated Hospital of Henan University of Traditional Chinese Medicine, Zhengzhou, China

**Keywords:** breast cancer, ultrasound, artificial intelligence, VOSviewer, CiteSpace

## Abstract

**Objective:**

Through bibliometric visualization analysis, this study aims to summarize research progress in artificial intelligence (AI)-integrated ultrasound technology for breast cancer, reveal research hotspots, development trends, and international collaboration patterns, thereby providing references for clinical diagnosis and therapeutic decision-making.

**Methods:**

Based on the Web of Science Core Collection (SCI-Expanded), we retrieved relevant literature from 2004-2025 (1,876 articles finally included). VOSviewer (v1.6.20), CiteSpace (v6.3.1 Basic), and Microsoft Excel 2019 were employed for visual analysis of publication volume, national/institutional collaboration, author networks, keywords, and co-citation relationships.

**Results:**

Annual publications have shown a progressive increase since 2024. The United States (485 articles, 15,394 total citations) demonstrated the highest academic influence. Core researchers included Moon Woo Kyung (38 articles), while Seoul National University Hospital (47 articles) emerged as a key collaborative institution. Keyword clustering identified “deep learning”, “breast ultrasound”, and “machine learning” as research hotspots, with burst detection analysis revealing “deep learning” as the most prominent emerging theme (post-2020 surge). Radiology ranked as the most cited journal (4,258 citations), with foundational works by Berg WA (2008) and Al-Dhabyani W (2020) constituting the highest-impact literature.

**Conclusion:**

AI-ultrasound integration is suggested to have potential for enhancing diagnostic accuracy in breast cancer, although global research still exhibits regional disparities. Future efforts should strengthen international collaboration, optimize deep learning-based imaging analysis, leverage big data for treatment optimization and prognosis prediction, while addressing technical challenges including data quality assurance and algorithm sharing mechanisms.

## 1 Introduction

Globally, breast cancer represents one of the leading malignancies among women, accounting for approximately 570,000 deaths in 2015 (1). A marked contrast exists in 5-year survival rates for breast cancer between high-income and low-to-middle-income countries, with the former typically demonstrating rates approximating 80%, while the latter generally fall below 40% (2). According to the American Cancer Society, 268,600 new breast cancer cases were diagnosed in the United States in 2019, with approximately 15% of patients succumbing to the disease. Concurrently, breast cancer incidence in China has demonstrated a consistent upward trajectory. Projections from the International Agency for Research on Cancer (IARC) indicate that, should current trends persist, global annual incident cases will rise to approximately 3.2 million by 2050 (3). Breast cancer risk factors can be categorized into two distinct classes: non-modifiable intrinsic variables such as sex, age, and ethnicity, along with hereditary determinants (e.g., BRCA1/2 mutations) and benign proliferative breast lesions; and modifiable extrinsic variables encompassing lifestyle choices (including alcohol consumption, obesity, and physical inactivity) as well as prolonged medical interventions (such as hormonal contraceptives or replacement therapy). While these factors may elevate breast cancer risk, implementation of early screening protocols and enhanced health education initiatives can effectively reduce both incidence and mortality rates (4). Research demonstrates that early-stage breast cancer patients achieve approximately 90% survival rates. Consequently, early detection, precise diagnosis, and timely intervention are critical for improving patient prognoses. Current clinical imaging modalities include conventional ultrasound, contrast-enhanced ultrasound, mammography, magnetic resonance imaging (MRI), and computed tomography (CT) (5–7). Among these, ultrasound has emerged as a preferred imaging modality in routine clinical screening and management of breast cancer, owing to its operational simplicity, non-invasive nature, radiation-free characteristics, and cost-effectiveness (8). However, ultrasonography remains limited by challenges such as low specificity and the operator-dependent nature of results, which may lead to potential variability in diagnostic accuracy (9). Addressing these limitations through standardized protocols and technological advancements urgently requires prioritized attention in clinical practice.

In recent years, the advent of artificial intelligence (AI) —a cornerstone general-purpose technology driving the new wave of scientific and industrial transformation — has rapidly permeated diverse domains. Notably, its integration into medical imaging has demonstrated significant potential in addressing the inherent limitations of conventional ultrasound practices, particularly through enhanced diagnostic precision and mitigation of operator variability (10, 11). (1) AI algorithms, including deep learning architectures such as U-Net and generative adversarial networks (GANs), effectively perform automated noise suppression in ultrasound images to enhance spatial resolution. (2) Establish AI models (such as Convolutional Neural Networks) achieve precise automatic annotation of pathological structures including nodules, thrombi, and masses (12). (3) AI systems enable automated biometric measurements (fetal head circumference, femur length) and congenital disorder screening (13, 14). The integration of AI with ultrasound technology is evolving from an “assistive tool” to a “decision-making system.” Furthermore, AI-driven ultrasound radiomics and high-throughput analysis substantially reduce the inherent operator dependency of conventional ultrasound techniques, holding promise for broader clinical adoption across diagnostic and prognostic workflows (15).

To deeply discuss the research hotspots and future prospects of ultrasound based on artificial intelligence in the application of breast cancer, and to provide a more valuable reference for subsequent clinical diagnosis and treatment decisions, this study innovatively employs two bibliometric software tools, VOSviewer and CiteSpace, to visualize articles in the field, uncovering the potential information behind the data.

## 2 Methods for data retrieval and usage

### 2.1 Sources and retrieval guidelines

**Figure 1:**
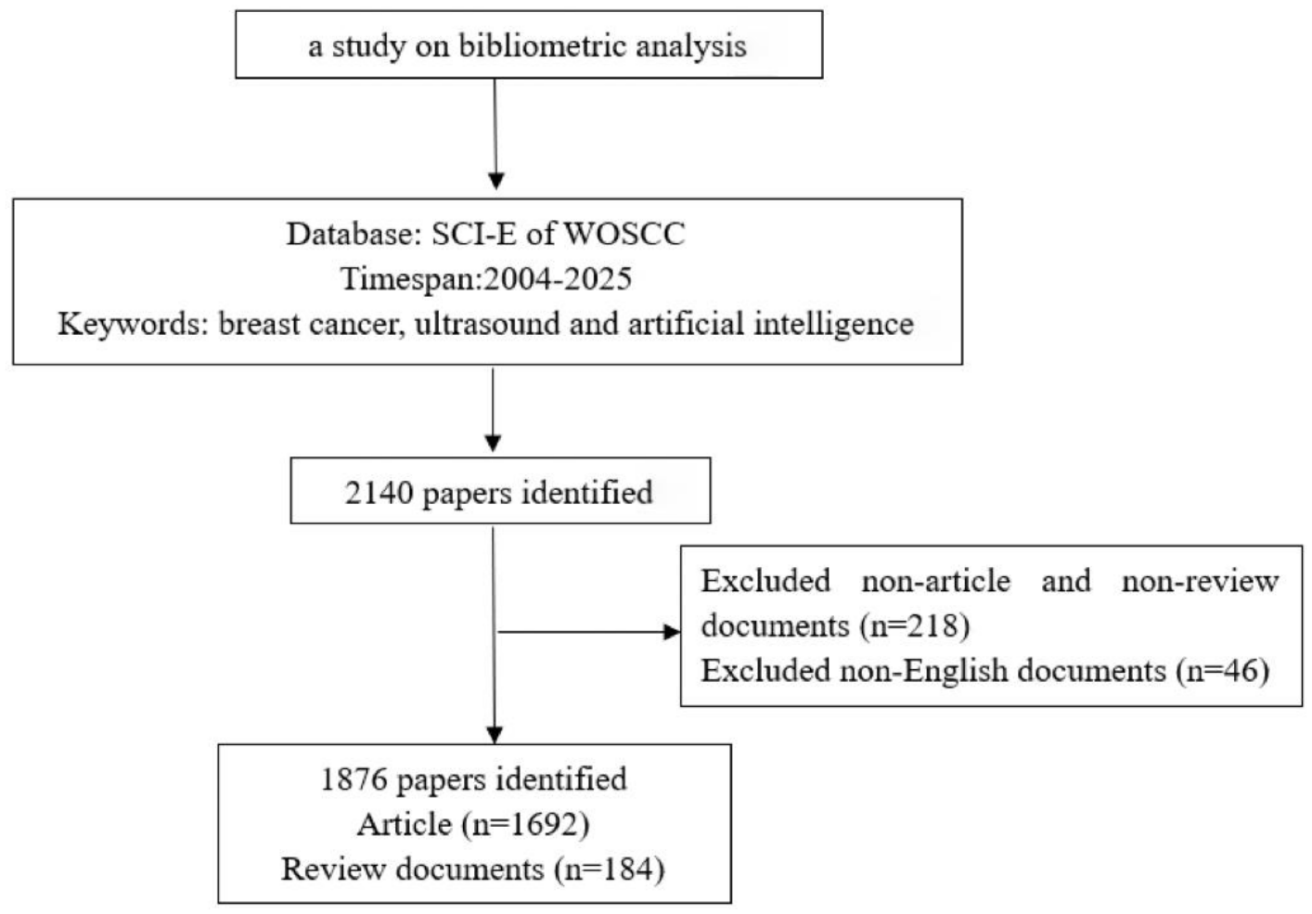
Flowchart of the study strategy.

Web of Science (WoS) (16), one of the largest academic literature databases globally, comprehensively indexes academic journals, conference proceedings, and doctoral/master’s theses across multidisciplinary research fields (17, 18). It is widely accepted by researchers as a high-quality digital bibliographic database and is considered the most suitable for bibliometric analysis (19). Therefore, this study chooses Web of Science as the literature retrieval platform, using the Science Citation Index Expanded (SCI-Expanded) version in the WoSCC database. The time range for retrieval is set from 2004 to present, with the keywords “breast cancer,” “ultrasound,” “Artificial intelligence,” and “breast ultrasound”. Inclusion criteria: English-language literature related to breast cancer, ultrasound, and artificial intelligence (AI); and those with complete bibliographic information (including title, country, authors, keywords, and source) (20, 21). Initial retrieval yielded 2,140 publications. Following the exclusion of non-article/non-review publications (n=218) and non-English records (n=46), a final dataset of 1,876 documents was obtained, comprising 1,692 articles and 184 reviews (see **Figure 1**). The exported records were subsequently processed for analysis and downloaded file has been renamed to “download_x.”

### 2.2 Overview of analytical tools

In the field of bibliometric and visual analytics, CiteSpace and VOSviewer (22)are widely utilized software platforms that employ specialized algorithms and visualization techniques to elucidate developmental trajectories and structural characteristics within research domains (23).

VOSviewer is a professional bibliometric software developed by Leiden University in the Netherlands. It enables the construction and visualization of scientific networks (e.g., journals, researchers, publications) based on citation, co-citation, co-authorship, or term co-occurrence relationships. The latest version (1.6.20, released October 31, 2023) features optimized data import functionalities, including support for Scopus’s updated file formats. The software is publicly accessible via its official website and requires a Java runtime environment for operation (24–26).

CiteSpace is jointly developed by Dr. Chaomei Chen from Ryerson University in the United States and the WISE Laboratory at Dalian University of Technology. Its powerful data analysis capabilities have been widely applied in various visualization fields. This study employed CiteSpace 6.3.1 Basic (valid from February 14, 2024, to December 31, 2025), which provides essential bibliometric analytical capabilities to identify research hotspots and emerging trends within a given field (26, 27).

## 3 Parameter configuration and results

### 3.1 Annual publication output analysis

**Figure 2:**
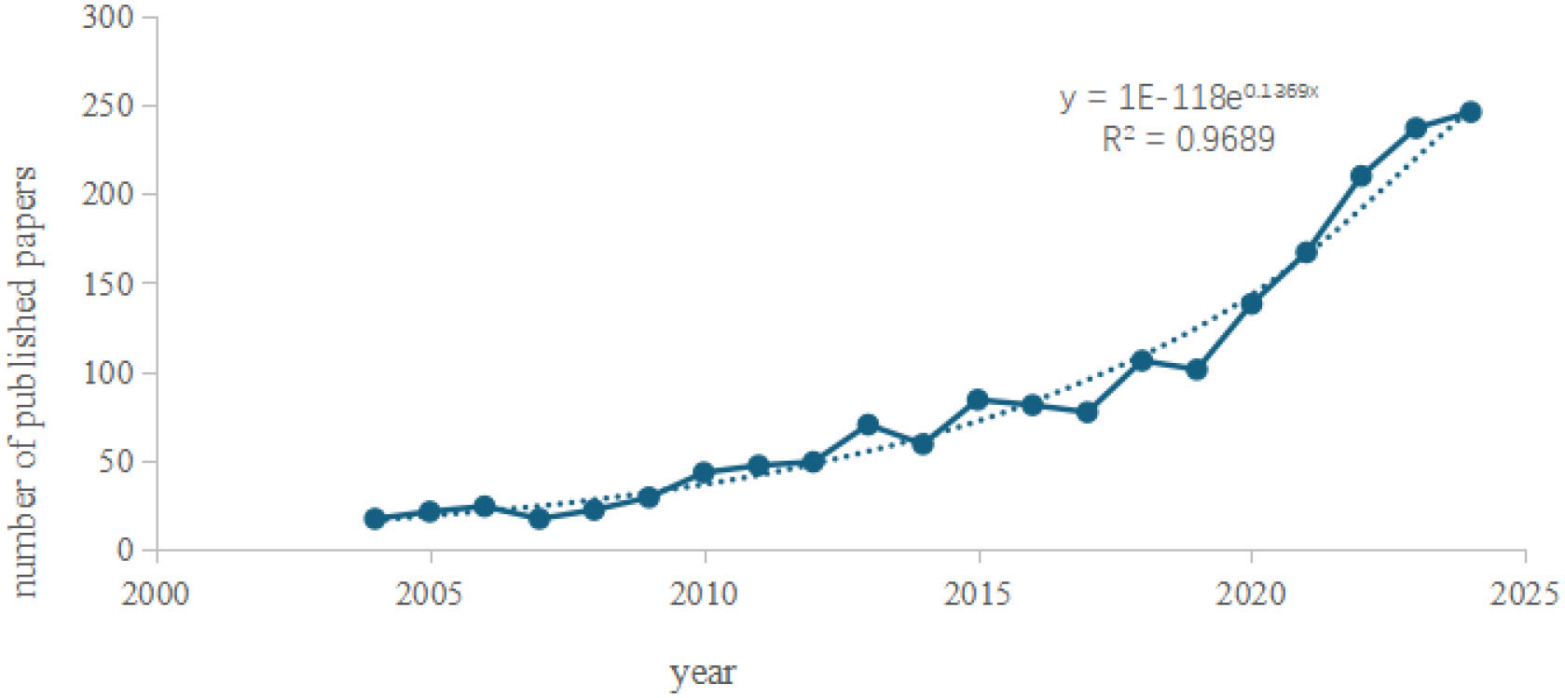
Annual publication volume trend chart.

Annual publication output serves as a critical metric for evaluating the progression of scientific research, reflecting the growth dynamics of a specific field. In this study, deduplication of literature records was performed using CiteSpace, followed by the importation of annual publication counts into Microsoft Excel 2019 for analysis via a two-dimensional line chart (**Figure 2**). The analysis reveals the annual publication trends in AI-integrated ultrasound research for breast cancer globally. Publications in this field emerged in 2004 and demonstrated a steady upward trajectory over time. Notably, a marked increase in output was observed between 2019 and 2025, with 2024 recording the highest annual publication volume to date. The coefficient of determination (R² = 0.9778) indicates a robust fit of the trendline, suggesting high statistical reliability. Based on this model, it is projected that publication output will continue to rise in 2025, reflecting escalating research activity. Furthermore, advancements in edge computing and 5G technology are anticipated to position AI-ultrasound interdisciplinary collaboration as a pivotal node in precision medicine and smart manufacturing.

### 3.2 Collaborative network

The analysis of collaborative relationships provides a new perspective on the mechanisms of cooperation between individuals and institutions (28). Co-authorship serves as a formal declaration indicating that a technical document results from the collective contributions of multiple authors or institutions. Despite ongoing academic debates regarding its precise definition and interpretation, co-authorship analysis remains an established methodology for evaluating and deconstructing scientific collaboration pattern (28, 29).

**Figure 3:**
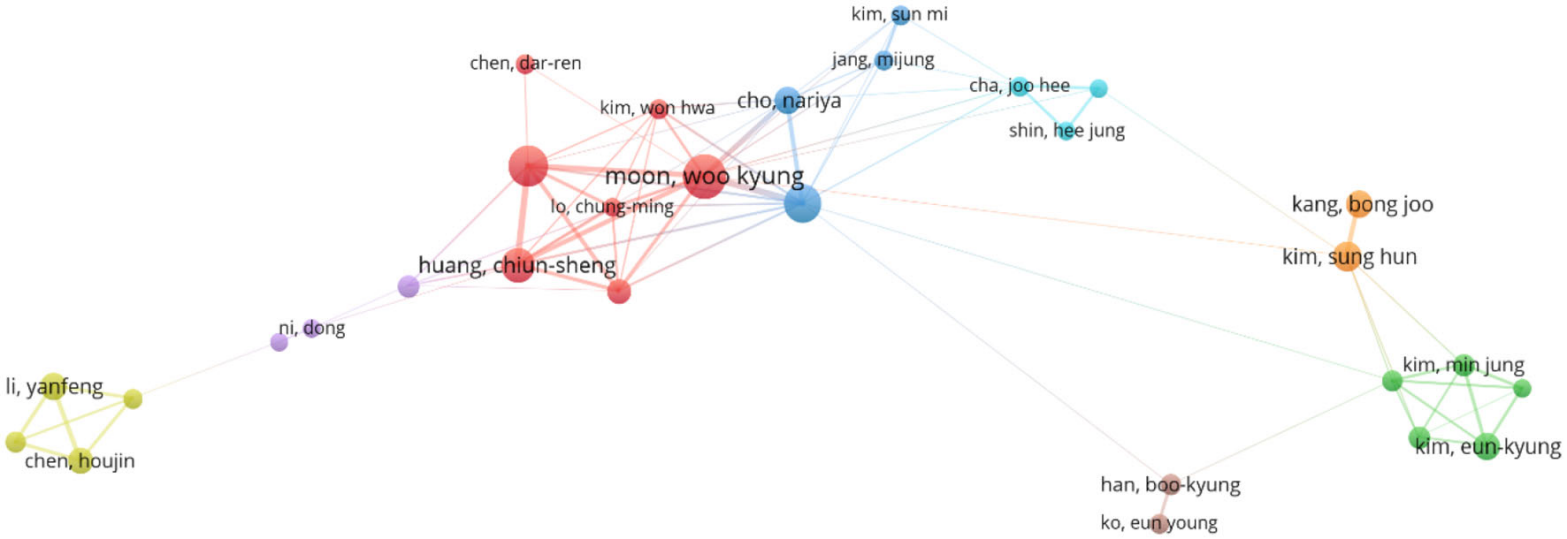
Co-author clustering view.

“Co-authorship clustering analysis” was performed using VOSviewer 1.6.19. The parameters were configured as follows: “Type of analysis” set to”Co-authorship”, “Unit of analysis” to “Authors”, and “Counting method” to “Full counting”, with a minimum publication threshold applied while retaining default settings for other parameters. The resulting author clustering network is illustrated in (**Figure 3**). Among 8,448 authors identified, 46 met the inclusion criterion of publishing at least nine articles. In terms of total link strength (TLS), Moon, Woo Kyung (38 publications, 1,489 citations, TLS = 104) exhibited the highest collaborative influence, followed by Chang, Ruey-Feng (32 publications, 1,126 citations, TLS = 80) and Huang, Chiun-Sheng (25 publications, 1,542 citations, TLS = 74). This visualization delineates collaborative networks among core contributors in AI-ultrasound research for breast cancer, providing a foundational framework for analyzing academic interactions and research team dynamics within the field.

**Figure 4:**
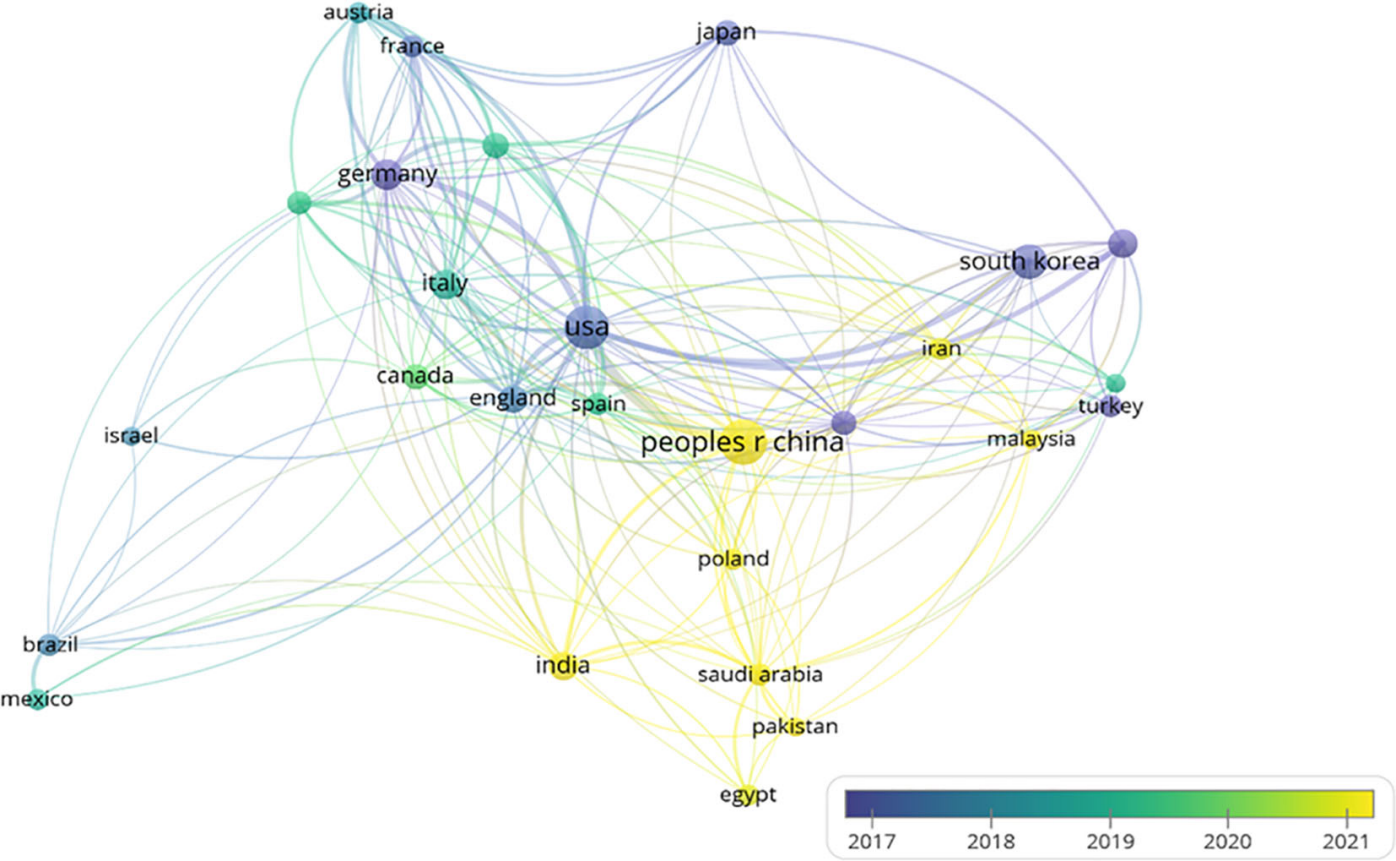
Country label view.

**Figure 5:**
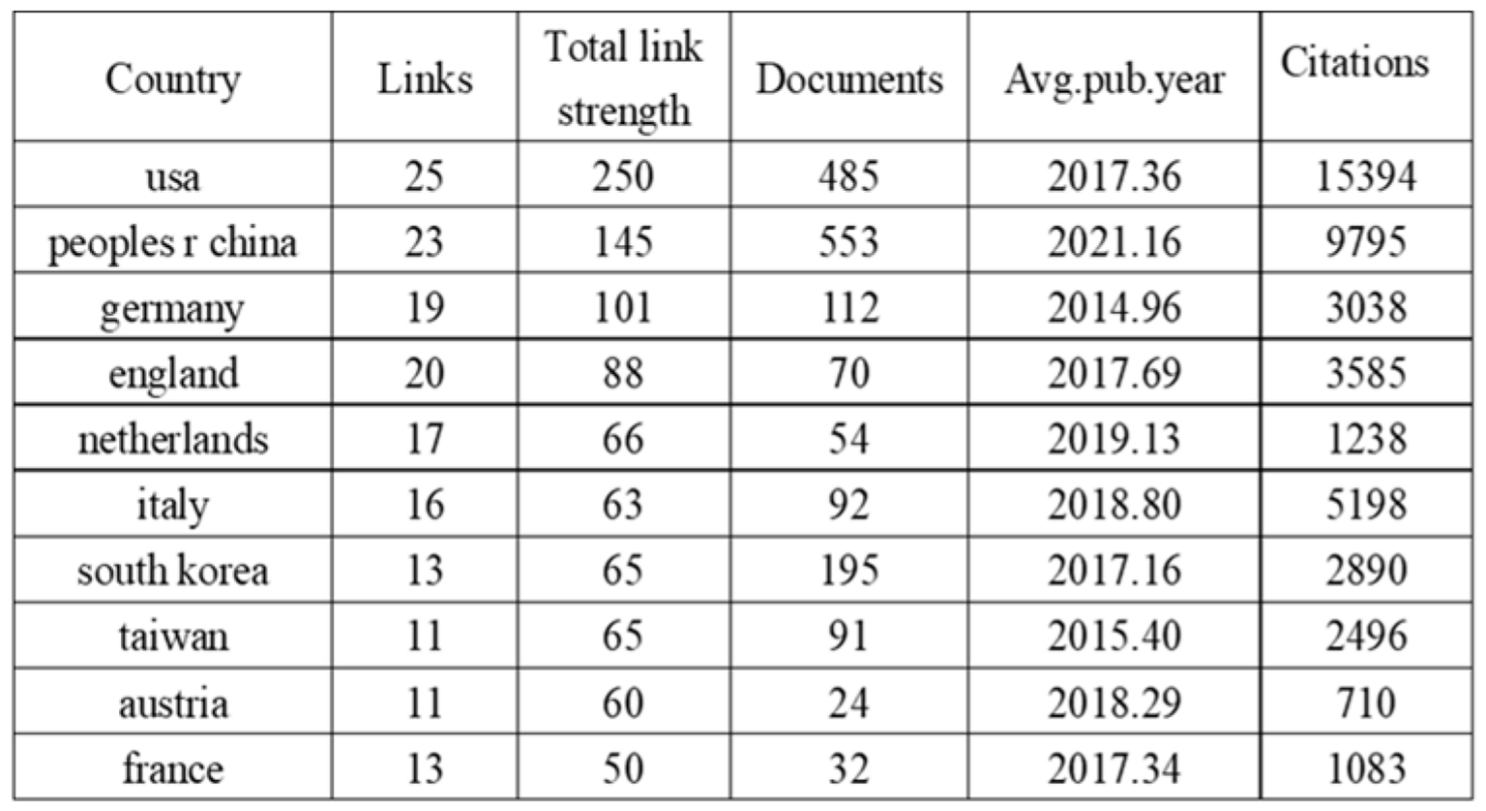
Top ten countries by publication volume.

The international collaboration network (**Figure 4**) comprises 83 countries, with 27 nations meeting the threshold of publishing at least 14 articles. Each circle represents a country, and the size of the circle reflects its intermediary centrality (centrality ≥ 0.1). As shown in (**Figure 5**) detailing the top 10 productive countries, the number of documents provided by USA is 485, with a total link strength of 250, an average publication year of 2017.36, and cited 15,394 times; the number of documents provided by Peoples R China is 553, with a total link strength of 145, an average publication year of 2021.16, and cited 9,795 times; the number of documents provided by Germany is 112, with a total link strength of 101, an average publication year of 2014.96, and cited 3,038 times. This indicates that the above three countries have a strong intention to cooperate.

**Figure 6:**
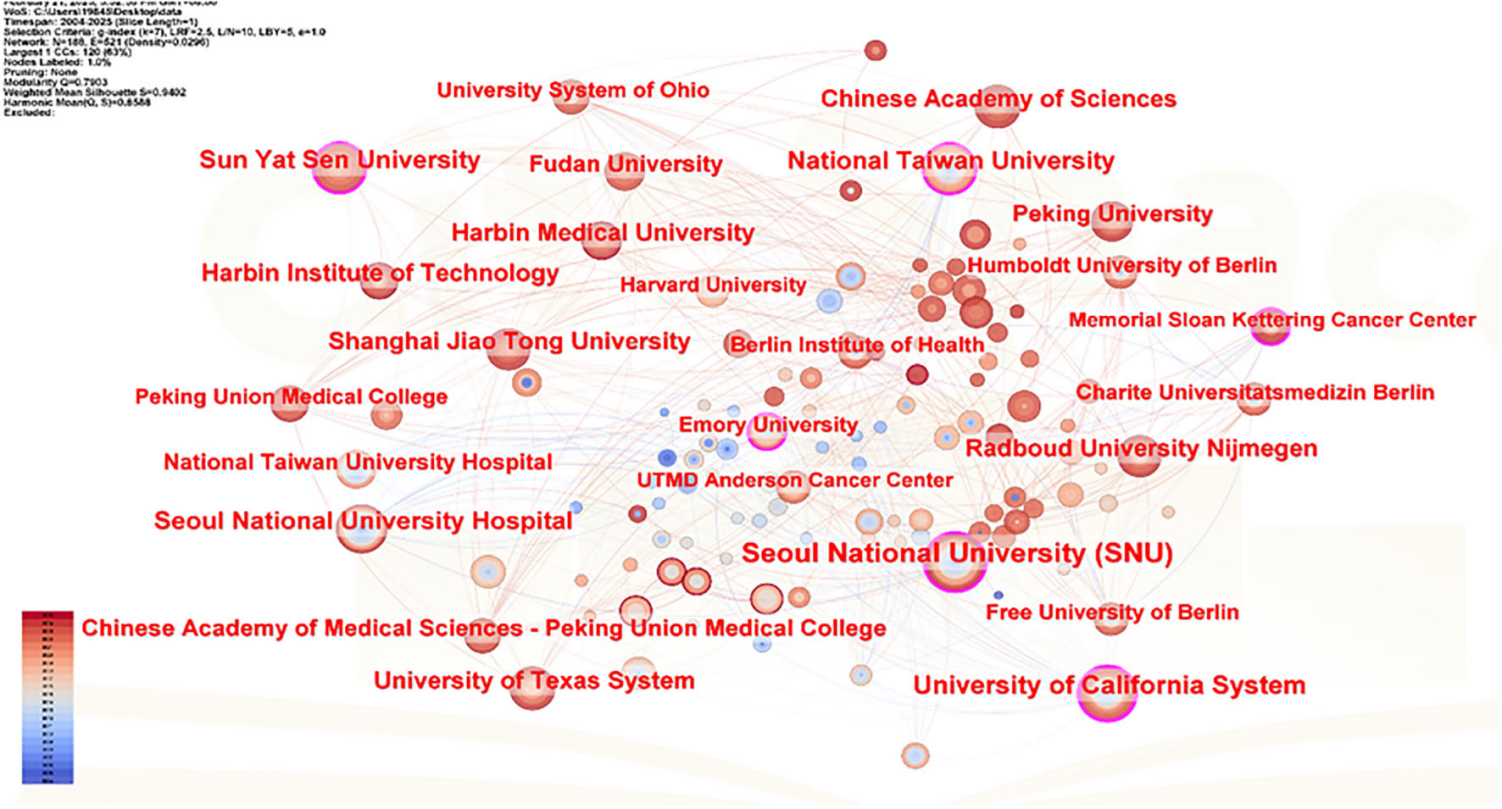
Institutional collaboration network view.

**Figure 7:**
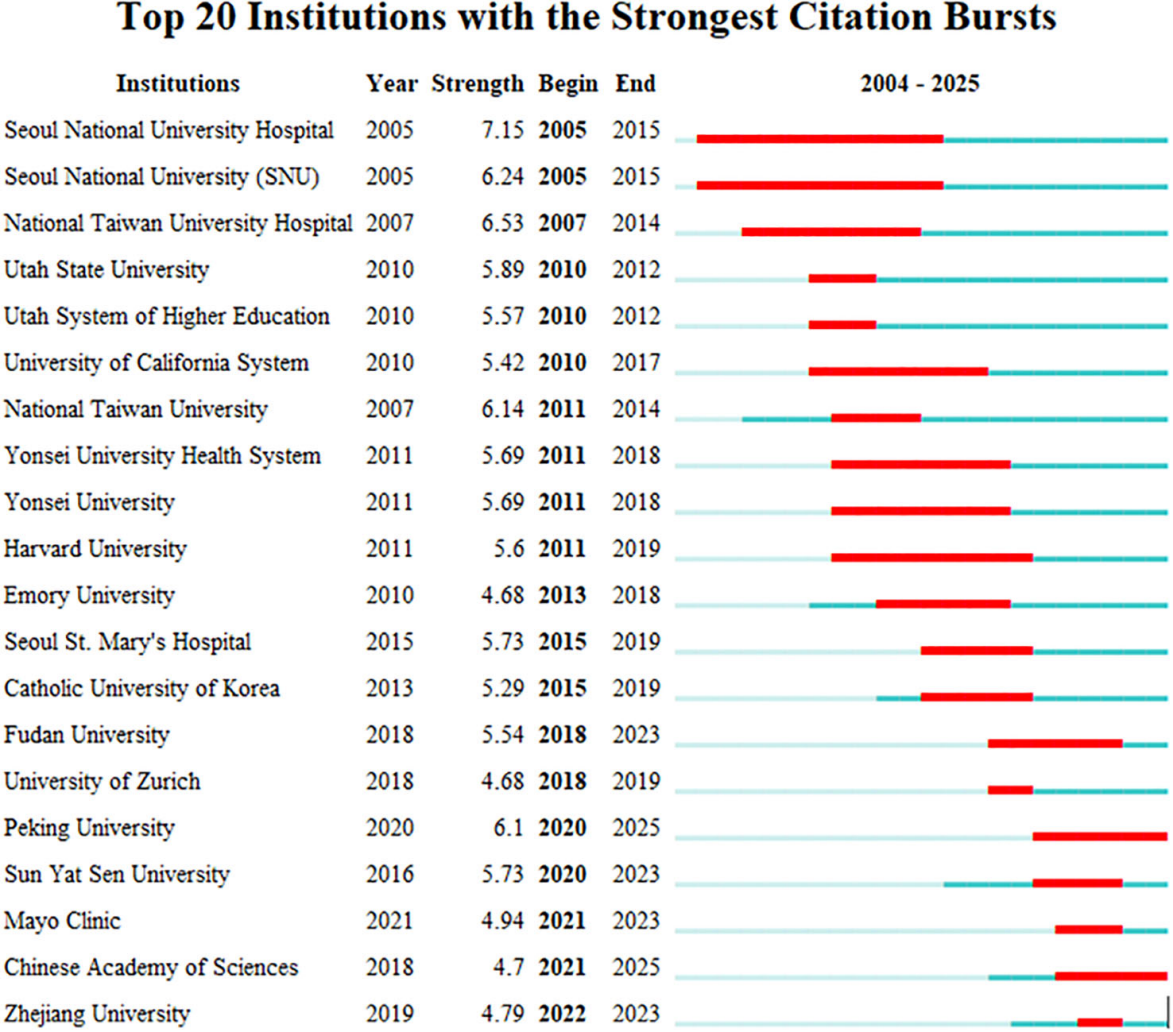
Institutional burst map.

In CiteSpace software, the “Plain text file” format literature exported from WOSSC was converted and named “Breast Cancer WOS.” This ultimately resulted in the generation of a collaboration network view (see **Figure 6**) and a burst keyword view (see **Figure 7**). In the maps, each node represents an institution, and the size of the node indicates the total number of papers published by that institution in the field. There are a total of 188 nodes and 521 edges, with a density of 0.0296, the Q value is 0.8588 and the weighted mean silhouette s is 0.9402. These values indicate that the clustering structure is clear, compact, and the results are reliable. Among these, the top three institutions in terms of publication output are Seoul National University Hospital (47 articles), National Taiwan University (47 articles), and National Taiwan University Hospital (32 articles). Regarding citation strength, the top five institutions are Seoul National University Hospital, Seoul National University (SNU), National Taiwan University Hospital, Utah State University, and the Utah System of Higher Education. These results demonstrate that these universities and their affiliated hospitals play a central role in the collaborative network of this field, maintaining close and active partnerships. Their collaborative efforts have significantly contributed to advancing the development and application of AI-assisted ultrasound technology in breast cancer research.

### 3.3 Keyword analysis

#### 3.3.1 Keyword co-occurrence

**Figure 8:**
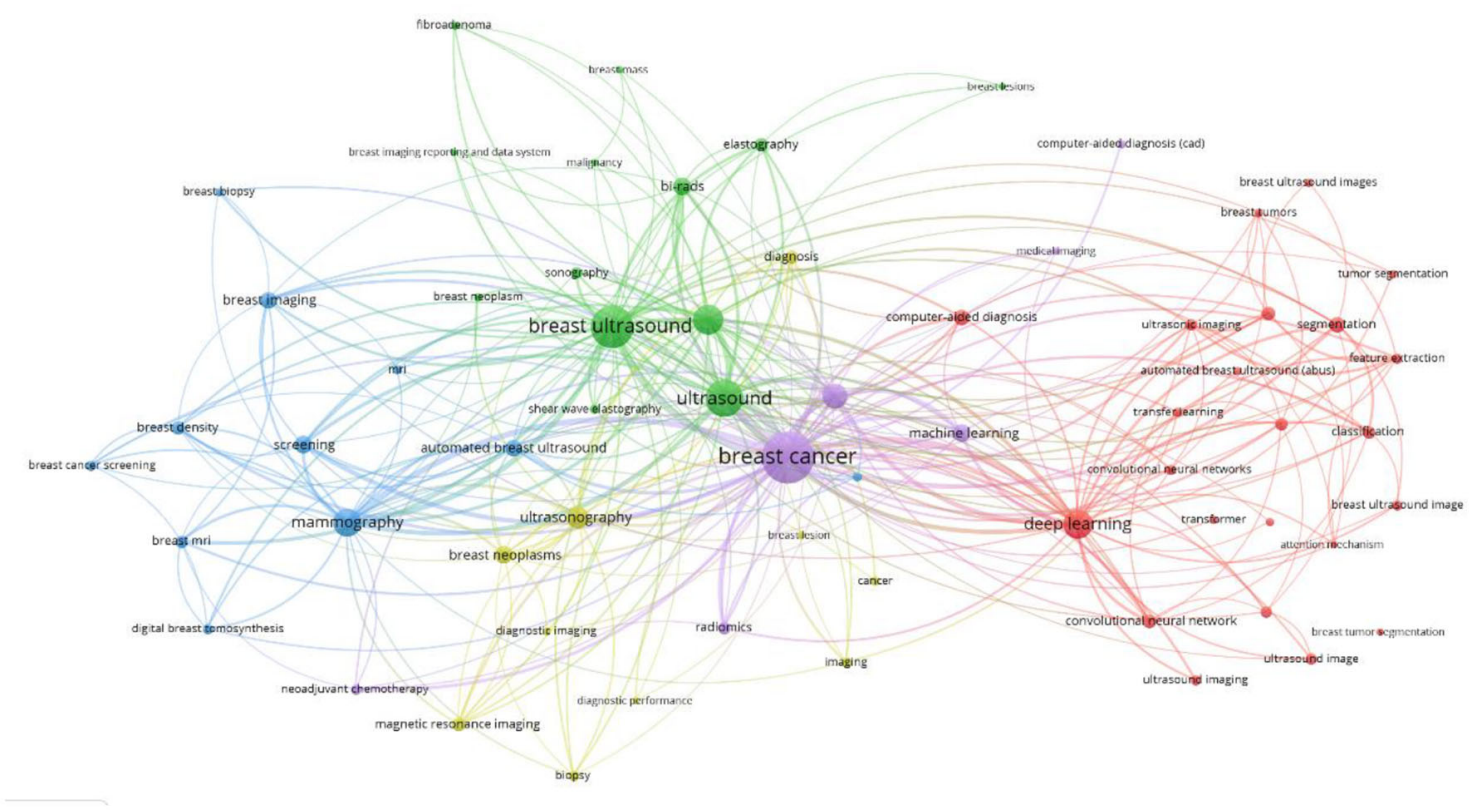
Keyword co-occurrence view.

**Figure 9:**
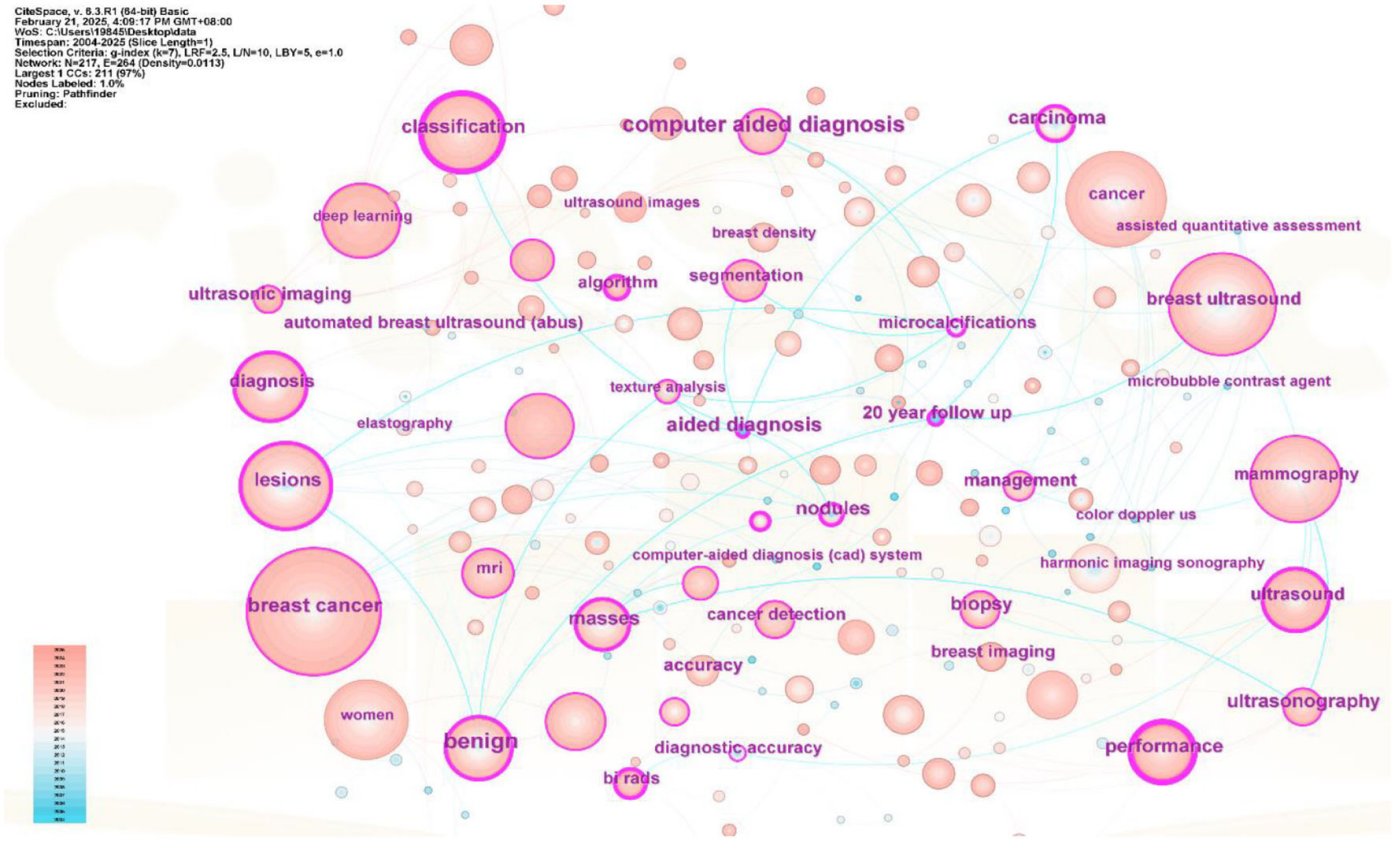
Keyword co-occurrence view.

Keyword network analysis can accurately link the development trends in the field and help precisely identify its hot issues and technological innovation (30). Using the VOSviewer 1.6.19 software, a total of 3,441 keywords were included, of which 64 reached the threshold of appearing at least 13 times (see **Figures 8**, **9** and **Table 1**). In CiteSpace, Per Slice was set to 1, with a time span from 2004 to 2025, and the node filtering method selected was g-index, with k set to 7 (See **Figure 9**).

**Table 1:** Distribution of the top 10 most frequent keywords in the published literature.

Serial number	Keyword	Occurrences	Total link strength
1	Breast cancer	585	1108
2	Breast ultrasound	399	592
3	Deep learning	191	478
4	Mammography	177	413
5	Ultrasound	299	399
6	Breast	201	367
7	Artificial intelligence	135	367
8	Machine learning	66	202
9	Ultrasonography	102	198
10	Screening	69	134

Different colors represent different clusters, each corresponding to a major research direction. These keywords are divided into five clusters (see **Figure 8**). The first cluster (red) mainly involves keywords such as deep learning, computer-aided diagnosis, and breast tumors; the second cluster (green) includes breast ultrasound, ultrasound, and bi-rads; the third cluster (blue) focuses on mammography, breast imaging, and breast MRI; the fourth cluster (yellow) revolves around ultrasonography, breast lesions, and breast neoplasms; and the fifth cluster (purple) encompasses breast cancer, machine learning, and artificial intelligence. Among these, breast cancer appears the most frequently, with a count of 585 and a total link strength of 1108. The frequencies for breast ultrasound, deep learning and mammography are also high, with specific data available in (**Table 1**).

**Figure 10:**
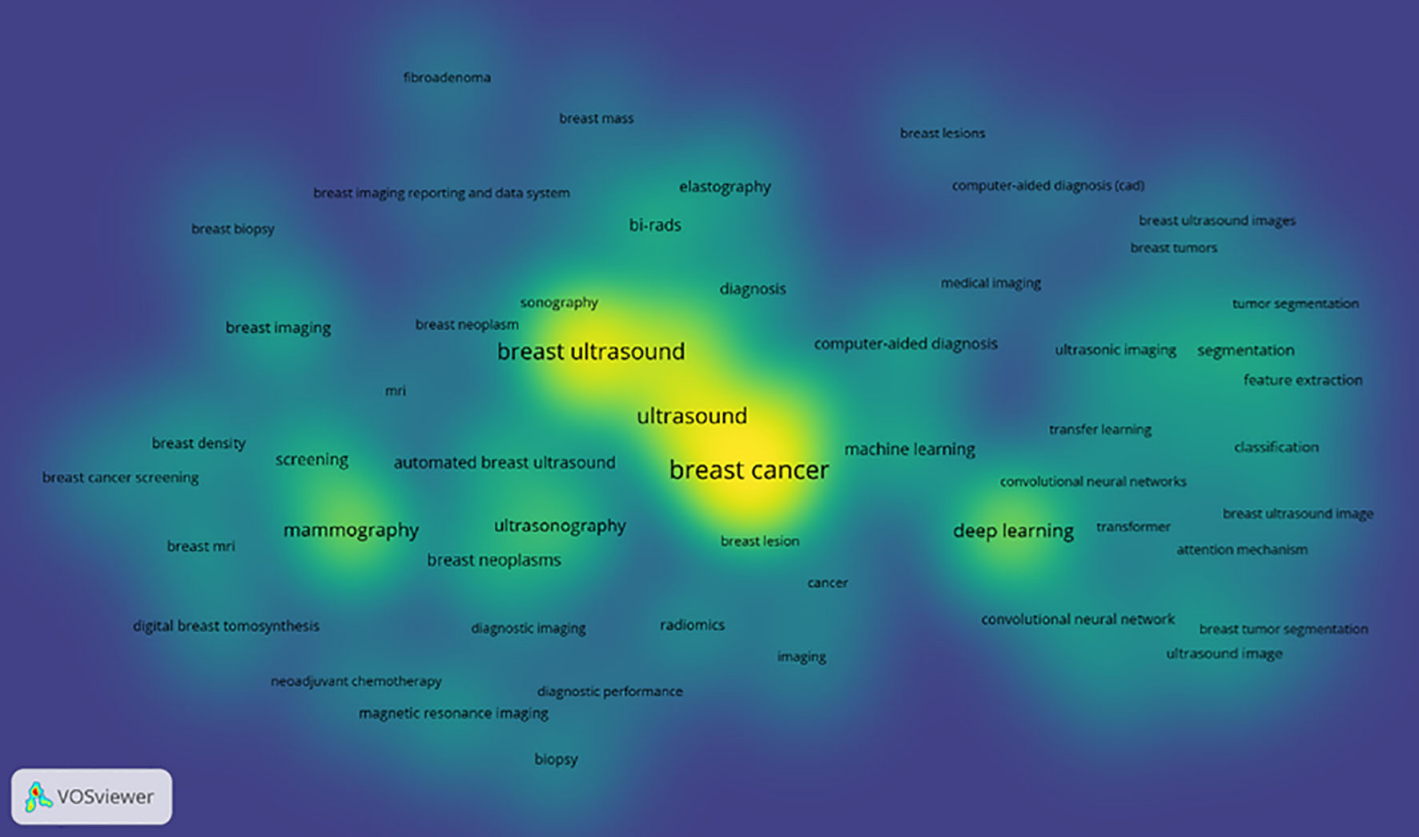
Keyword density co-occurrence view.

The keyword density view (see **Figure 10**) presents the density of keywords using color coding, where the color variation reflects the frequency or density differences of the keywords. A higher density corresponds to colors closer to yellow, while a lower density corresponds to colors closer to blue. This aids in quickly identifying the distribution of research hotspots or important areas.

Excluding search keywords related to “breast cancer,” “breast ultrasound,” and “ultrasound,” the term “classification” is the most central, with a score of 0.53 and appearing 221 times, Analyzing the keywords with high centrality and frequency reveals that the main focus is concentrated on the treatment of breast cancer, involving “computer-aided detection” “aided diagnosis” and “bi rads” with additional keywords such as “algorithm” “microcalcifications” and “mammography”, A centrality greater than 0.1 indicates that these keywords are extremely important in the research field and represent major research hotspots.

#### 3.3.2 Keyword clustering

**Figure 11:**
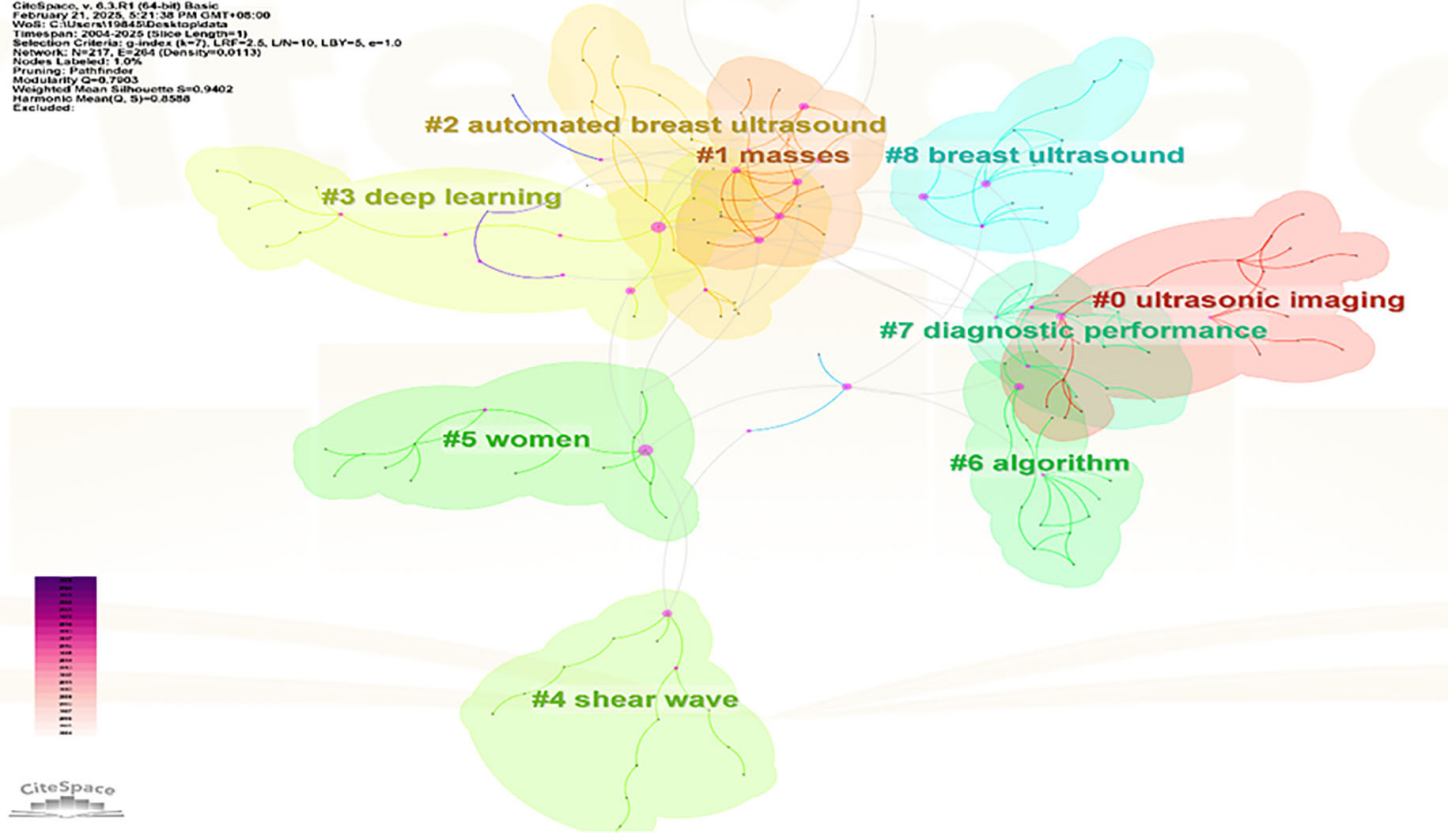
Keyword clustering view.

Apply the LST algorithm to perform cluster analysis on the identified keywords, resulting in a modularity Q value of 0.7903, that confirms the reliability of the clustering structure. A total of 9 clusters were formed, the specific tags are as follows.: #0 ultrasonic imaging, #1 masses, #2 automated breast ultrasound, #3 deep learning, #4 shear wave, #5 women, #6 algorithm, #7 diagnostic performance, and #8 breast ultrasound (See **Figure 11**).

#### 3.3.3 Keyword emergence

**Figure 12:**
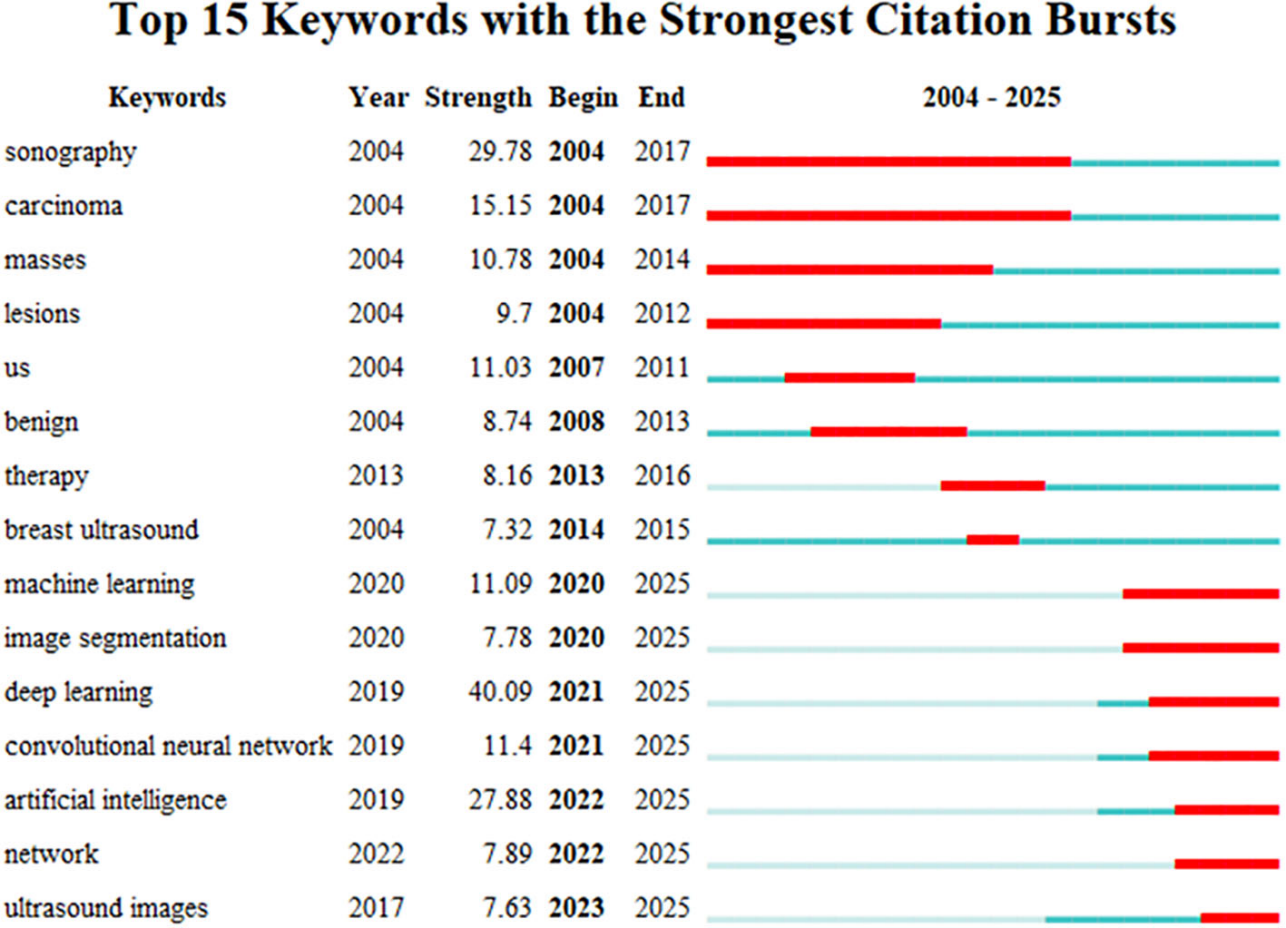
Keyword burst map.

A “surge word” refers to a keyword that experiences a sharp increase in frequency during a specific period, representing the development trends and future hotspots of a research field (31). A chart was generated through data analysis of emerging words, ranking the top 15 keywords according to their prominence strength (See **Figure 12**). In the chart, each line represents a period, and the red band indicates the period during which the hot search for that keyword experienced a sharp increase. The emergence of terms began in 2004, indicating that the application of AI in the area of breast cancer has garnered over twenty years of attention. The graphical representation demonstrates that deep learning currently exhibits the highest methodological prominence among artificial intelligence approaches, suggesting a sustained trajectory toward continued evolution and refinement of deep neural network architectures in AI-driven breast cancer research. It also fully demonstrates that the application of ultrasound in breast cancer is in a dynamic development process, reflecting the continuous changes in societal demands and shifts in research hotspots. At this stage, the combination of artificial intelligence and ultrasound provides more precise technical support for the diagnosis and treatment of breast cancer, becoming an important development trend in this field.

#### 3.3.4 Keyword time zone

**Figure 13:**
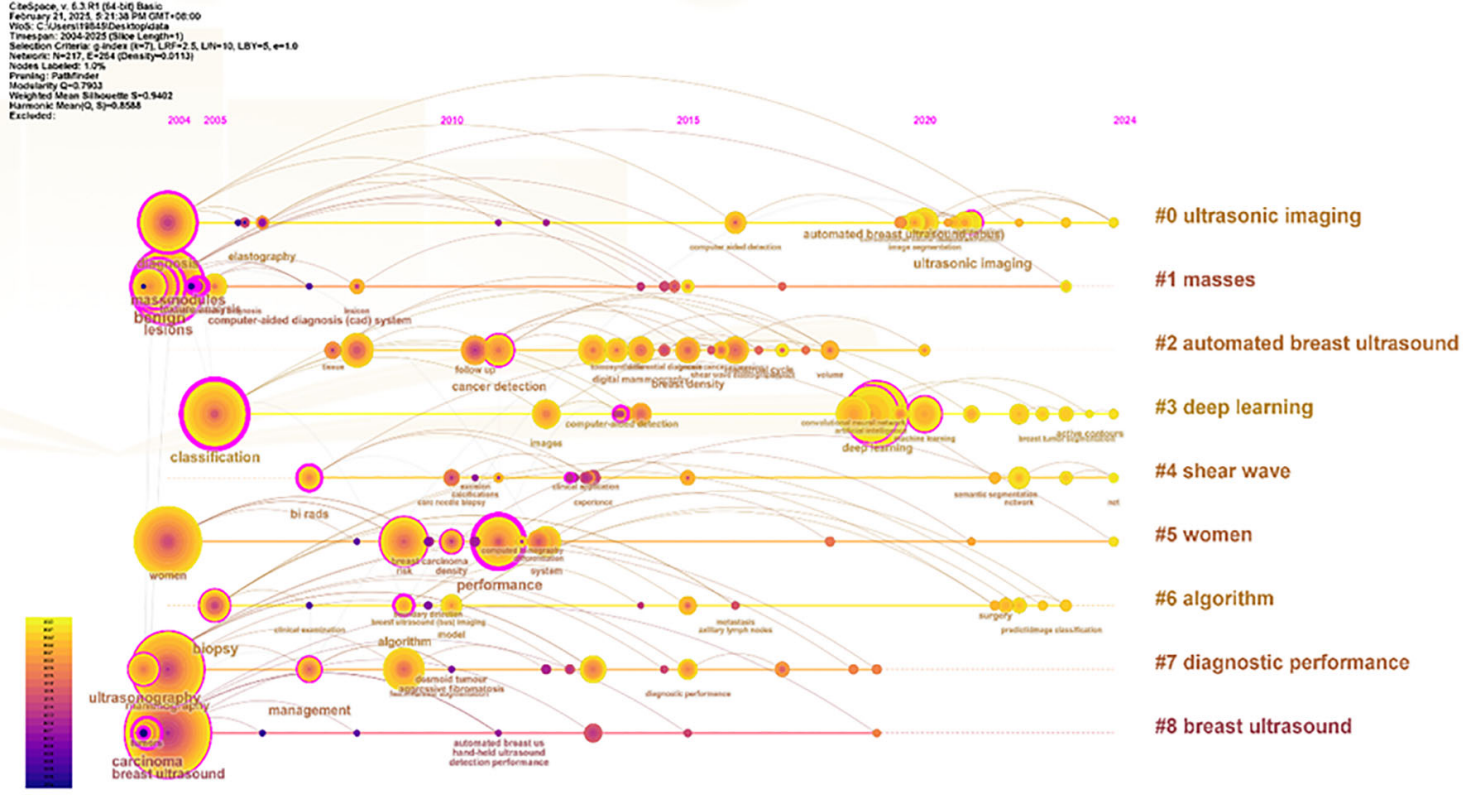
Keyword timeline view.

The size of each node reflects the intensity of focus on research trends each year, while the connecting lines show how research hotspots change over time. Generated nine clusters (See **Figure 13**), Among them, #4 shear wave and #6 algorithm are the most concentrated keywords. The first keywords to appear are #0 ultrasonic imaging, #1 masses, #5 women, #7 diagnostic performance, and #8 breast ultrasound. Over time, the most active and trending keywords in 2024 will be #0 ultrasonic imaging, #3 deep learning, #4 shear wave, and #5 women, representing the rapid development in the field of artificial intelligence and its critically important role in healthcare.

### 3.4 Co-citation analysis

#### 3.4.1 Analysis of co-cited references

**Figure 14:**
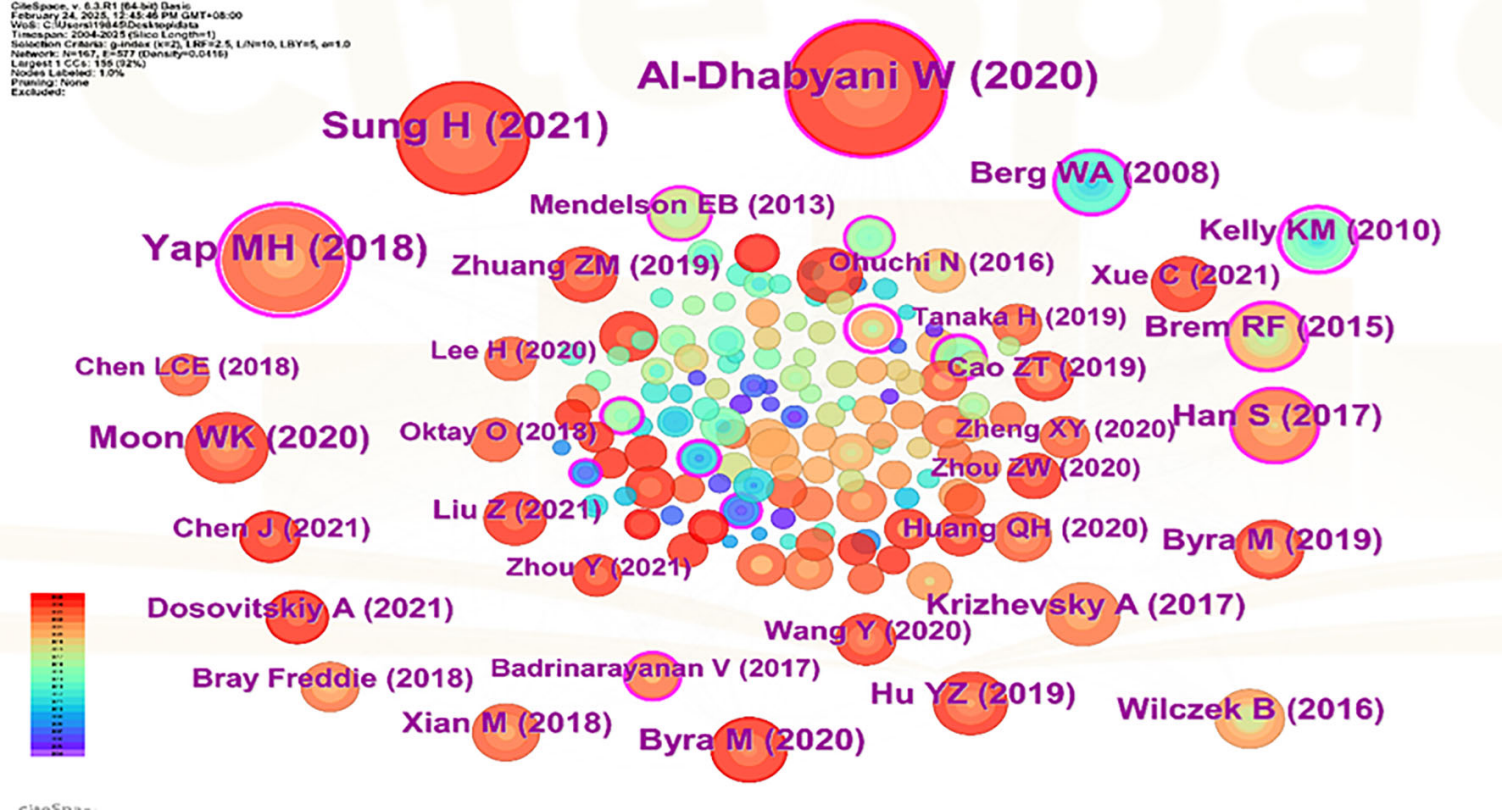
Co-Citation network view of references.

**Figure 15:**
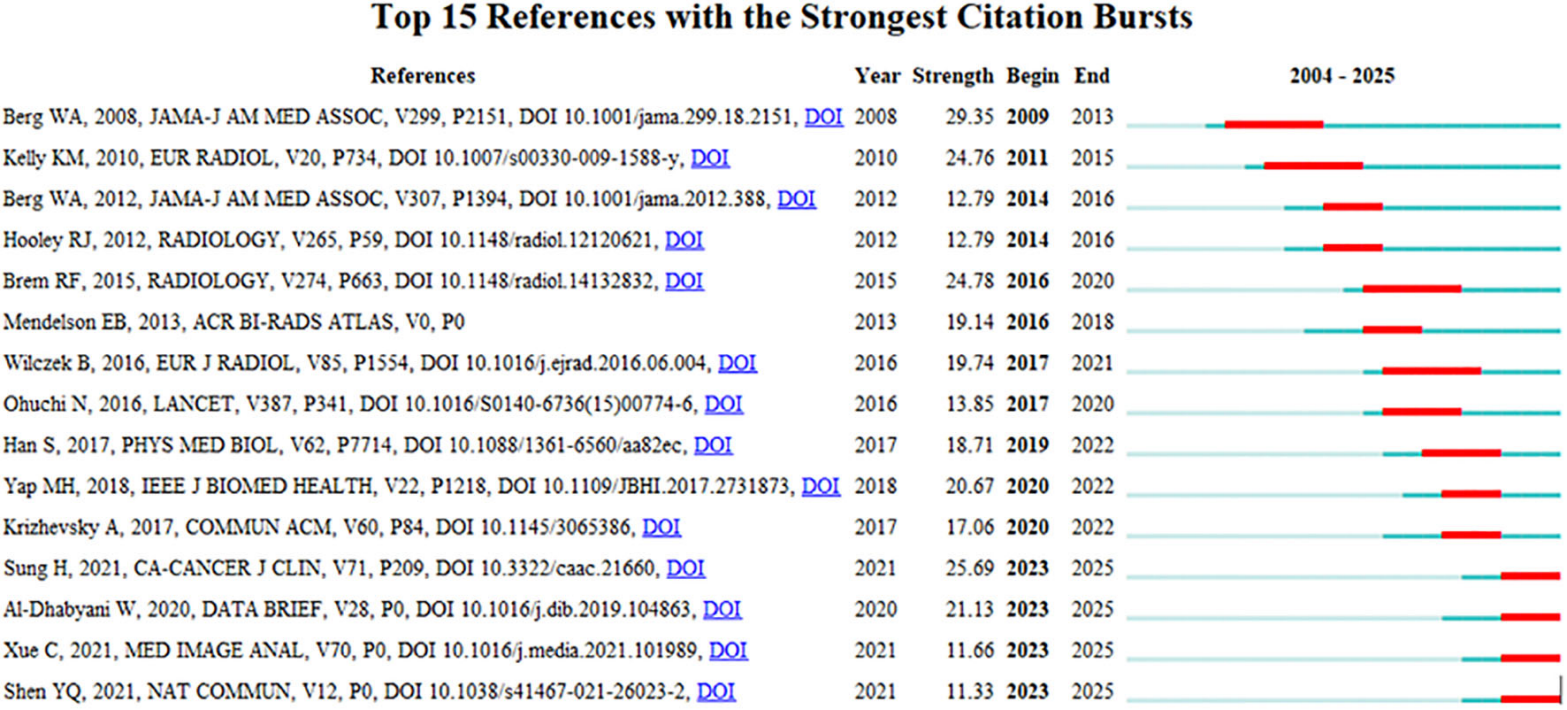
CiteSpace visualization map of top 15 references with the strongest citation bursts from 2004 to 2025.

Visual analysis was conducted using VOSviewer and CiteSpace software, incorporating a total of 39,883 reference articles. Among these, 59 articles were cited at least 47 times. Notably, the article by Berg WA, 2008, published in JAMA-J AM MED ASSOC, V299, P2151, DOI 10.1001/jama.299.18.2151, was cited 208 times, while the article by Al-Dhabyani W, 2020, published in DATA BRIEF, V28, P0, DOI 10.1016/j.dib.2019.104863, was cited 205 times. This analytical finding suggests that the two aforementioned references demonstrate the highest scholarly impact and academic value within social media platforms and digital communication networks. The density is 0.0416 (see **Figure 14**), indicating that the citations in this literature have a prominent clustering effect and a strong network homogeneity. **Figure 15** delineates the fifteen most frequently cited scholarly works in this domain, with bibliometric analysis revealing a marked escalation in citation rates commencing in 2009.

#### 3.4.2 Journal co-citation analysis

**Figure 16:**
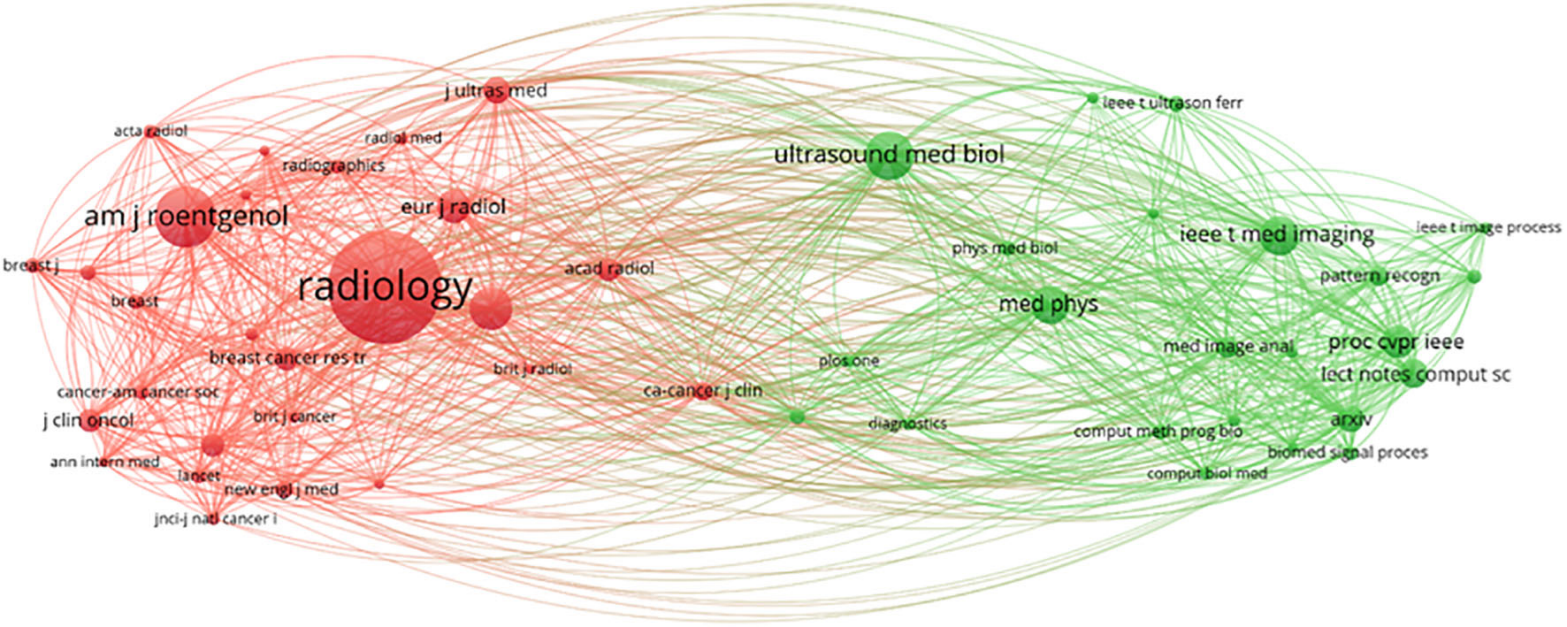
Journal co-citation network view.

A visual analysis was conducted using VOSviewer, which included a total of 9,099 journal sources. Among these, 50 articles were cited at least 300 times (see **Figure 16**). The journal “Radiology” was cited 4,358 times, “AJR American Journal of Roentgenology” was cited 2,212 times, and “European Radiology” was cited 1,472 times, indicating that these three types of journals have the highest international recognition. There are two clusters in total. The first cluster (red) includes journals such as “Radiology,” “AJR American Journal of Roentgenology,” and others. The second cluster (green) encompasses journals like “Ultrasound in Medicine & Biology,” “Medical Physics,” “IEEE Transactions on Medical Imaging,” among others (32).

## 4 Results

Over the past 20 years, artificial intelligence (AI) has seen rapid development, with most medical specialties engaging in exploration for clinical application (33). Compared to traditional systematic reviews, bibliometric analysis is a quantitative method for assessing and mapping scientific literature, providing a powerful tool for comprehensively studying research trends, influential contributors, and emerging topics in a specific field (34). This is the first attempt to use the bibliometric software VOSviewer and CiteSpace to summarize the current application of artificial intelligence-based color Doppler ultrasound in the field of breast cancer, as well as to visually predict its development trends and future research hotspots.

With the changes of the times and advancements in technology, the number of global submissions has been increasing year by year (**Figure 5**) and is expected to continue rising steadily in the future. The People’s Republic of China holds a leading position in the global research output in this field (as shown in **Figure 5**). Despite the People’s Republic of China showcasing a significant research productivity advantage with 12,487 publications (accounting for 28.3% of the global total), its overall citation impact (9,795 citations) reveals a gap of 36.4% compared to the U.S.-led research coalition (15,394 citations). This disparity in citation performance highlights the differentiated patterns of international collaboration networks and research priorities, manifested in U.S. institutions having closer cross-border collaboration relationships. The primary reason for this situation is that People’s China has a relatively late start in artificial intelligence technology, with an average publication volume of 2021.16. Following closely is the USA, which has the highest citation count and overall link strength, demonstrating that the USA possesses greater influence globally and collaborates extensively with other countries; The analysis of the top ten countries and the top twenty institutions in terms of publication volume shows that, with the exception of People’s China, most are developed nations. This indicates that developing countries are significantly lagging behind developed countries in the use and research of artificial intelligence ultrasound technology in breast cancer. The analysis further indicates that developed nations demonstrate heightened prioritization of breast cancer prevention and control, with widespread societal emphasis on screening, diagnosis, and treatment initiatives. Supported by funding and policy frameworks from national health authorities, advanced technologies are increasingly being implemented to enhance early detection and precision treatment of breast cancer. Therefore, we believe that China should actively maintain multilateral cooperation relationships and systematically attract cutting-edge global innovations to enhance its research competitiveness in emerging fields such as artificial intelligence. To promote international collaboration in artificial intelligence and ultrasound integration technology, we suggest the establishment of a specialized international research platform by global authoritative research institutions. This platform would integrate resources, share data, and research findings to facilitate the dissemination and exchange of knowledge. Although this study was restricted to English-language literature from the Web of Science Core Collection (WoSCC), potentially overlooking significant contributions from non-English publications and diverse geographic regions, these limitations are deemed methodologically justifiable given the current research scope and prevailing thematic trends. In subsequent investigations, we will undertake systematic integration of multilingual, multi-platform scholarly resources to enable comprehensive cross-linguistic comparative analysis, thereby enhancing the analytical robustness and scientific validity of our findings.

Moreover, the analysis by co-authors shows (**Figure 3**) that most authors have a BC value generally below 0.1, indicating that despite the involvement of numerous authoritative scholars in this research, they remain relatively disconnected. Moon and Woo Kyung have published a significant number of articles, showcasing their substantial impact in this field. However, despite the existence of many researchers in this area, there is a lack of stable connections with other researchers and institutions. It is suggested that stronger collaborations among authors be encouraged to expand research perspectives. As shown in (**Figure 8**), all keywords are categorized into five groups: “deep learning,” “breast ultrasound,” “mammography,” “ultrasonography,” and “machine learning.” The emergence of “deep learning” marks a transformation in medical imaging and introduces an innovative paradigm for interpreting radiological images (35), As a critical subfield of machine learning, deep learning centers on artificial neural networks with representation learning capabilities. These architectures automatically extract features via training on extensive datasets while enabling flexible decision-making aligned with task objectives. Kallenberg and others were the first to apply deep learning techniques in breast density evaluation (36). Zhang et al. developed a deep learning architecture for differentiating benign versus malignant breast tumors using shear wave elastography (SWE) images. Evaluated on a dataset comprising 135 benign and 92 malignant cases, the model demonstrated favorable classification performance with an accuracy of 93.4%, sensitivity of 88.6%, specificity of 97.1%, and area under the curve (AUC) of 0.947 (37). Coronado-Gutiérrez et al. developed a deep learning-based quantitative ultrasound (QUS) analysis method for noninvasive assessment of axillary lymph node involvement in breast cancer patients. Utilizing a dataset of 118 lymph node ultrasound images, the approach achieved a diagnostic accuracy of 86.4%, with sensitivity and specificity of 84.9% and 87.7%, respectively (38).

According to the annual publication volume (**Figure 12**), the analysis identifies two main periods: the first period, from 2004 to 2017, primarily focused on the domains of medical imaging, oncology, and pathology, specifically on topics such as “sonographs,” “carcinoma,” and “masses,” along with other related subjects. The second period, from 2020 to 2025, mainly centers on collaborations in artificial intelligence, machine learning, and deep networks, emphasizing research in areas such as “convolutional neural networks,” “machine learning,” “artificial intelligence,” and “image segmentation,” at this stage, deeper technologies of AI are beginning to take effect. Innovations in machine learning, image segmentation, and breast ultrasound imaging recognition provide possibilities for the treatment, screening, and monitoring of breast cancer (39). Ratnakar Dash et al. developed a customized Convolutional Neural Network (CNN) model for analyzing multimodal breast images. This framework autonomously extracts image features and classifies them into three categories: normal, benign, and malignant. Validation on standardized benchmarking datasets demonstrated classification accuracies of 97.45%, 96.30%, 98.80%, 99.25%, and 99.97%, confirming the model’s efficacy and superior performance in processing multimodal datasets (40). In recent years, Transformer-based architectures have demonstrated substantial potential for medical applications. Originally developed for natural language processing, these models have transitioned to multimodal domains including medical imaging and temporal data, exhibiting exceptional modeling capacity and robust generalization capabilities. The Vision Transformer (ViT) models global contextual information through self-attention mechanisms, enabling more effective capture of long-range dependencies in mammographic images. This architecture demonstrates particular strengths in detecting subtle pathological features such as microcalcifications and masses, thereby enhancing early breast cancer detection capabilities. In a systematic investigation, Yuan, Boyao et al. evaluated the performance of mainstream deep learning architectures—including CNN and Transformer models—for breast cancer histopathological image classification across tasks ranging from binary to octonary classification. The results indicate that CNN models exhibit superior performance in simpler tasks, potentially attributable to their stronger inductive bias and localized feature extraction capabilities. For octonary classification tasks, performance disparities among models become more pronounced, with the fine-tuned UNI model demonstrating optimal overall performance. Despite architectural differences, both paradigms exhibit robust image classification abilities, where performance variations are primarily attributable to task complexity and model-feature compatibility (41).

The keyword “deep learning” (strength=40.09) shows the highest intensity of emergence, starting from 2021, the future will continue to play an increasingly important role. Another significant citation surge is related to the keyword “sonography” (strength=29.78), with a time span from 2004 to 2017. Particularly noteworthy is the keyword “artificial intelligence” (strength=27.88), which has maintained dominance from 2022 to the present. These surges indicate that the position of artificial intelligence technology in the healthcare sector will gradually enhance. As an efficient, precise, and robust tool, deep learning can effectively alleviate clinical workloads. In breast ultrasound image analysis, this technology demonstrates significant potential for clinical translation across domains including image classification, object detection, and segmentation. Consequently, understanding its fundamental principles is essential, while potential implementation challenges require systematic consideration with proactive solution development (42).

Journals are essential tools for disseminating research findings and innovative ideas, and their quality and reputation play a crucial role in advancing scientific progress and human development (43). The journals being cited can be divided into two main categories (**Figure 16**), with the top three being “Radiology” (IF = 12.1, Q1), “AJR American Journal of Roentgenology” (IF = 4.7, Q1), and “European Radiology” (IF = 3.2, Q1). Impact Factor, JCR categories, and total citation counts are key indicators of journal quality. Additionally, the total citation count of “Radiology” far exceeds that of other publishing journals. It has verified the journal’s pivotal position in the subject area and expected that more research on the application of AI ultrasound in breast cancer will be prioritized for publication in the above-mentioned journals in the future.

The changes at the forefront of research can be demonstrated through co-cited references. Although these cited publications represent early-stage research with potentially limited contemporary innovation due to their publication dates, their enduring scholarly authority and substantive quality result in collectively constrained yet persistent academic influence. According to the citation analysis (**Figure 15**), this phenomenon first erupted in 2009, demonstrating AI-ultrasound integration in breast oncology has initiated within the past decade, with numerous references still being frequently cited. Throughout the entire research analysis, the application of artificial intelligence combined with ultrasound technology in breast cancer has undergone two significant phases. In the early stage, the focus was primarily on basic medical imaging techniques, pathological research, and oncology, while also intertwining the risk factors and prognostic diagnosis of breast cancer. Recently, however, there has been an integration of deep learning, machine learning, and convolutional neural networks, which are now regarded as research hotspots and represent the latest frontiers in breast cancer screening, diagnosis, treatment, and prognosis. Kiran evaluated multiple clinically deployed deep learning models, including VGG16, VGG19, and Alex Net, and introduced a novel hybrid architecture termed “EfficientKNN”. This framework integrates EfficientNetB3’s high-efficiency feature extraction capabilities with the computational simplicity and efficacy of k-nearest neighbors (k-NN) algorithms. Through foundational model optimization, EfficientKNN demonstrated superior performance in diagnostic accuracy and validation loss minimization, exhibiting both high classification precision and robust clinical utility (44). Adyasha Sah developed an efficient deep learning-based breast cancer detection system that integrates the strengths of AlexNet, ResNet, and MobileNetV2 architectures to enhance diagnostic performance. This framework is specifically designed for identifying abnormal tissues and malignant lesions, achieving 97.75% accuracy in malignancy detection tasks. The model demonstrates exceptional classification capabilities with robust adaptability across multimodal breast imaging datasets (45).

Driven by the dual forces of changing times and technological innovation, it has demonstrated excellent performance and high precision under laboratory conditions, capable of optimizing the care of cancer patients and bringing transformative changes to the field of oncology on a larger scale (46). Wang developed a novel weakly supervised two-stage detection and diagnosis network (TSDDNet). This model demonstrates state-of-the-art performance in both lesion detection and diagnosis tasks, highlighting its significant application potential in medical image analysis (47). Ren et al. developed DLMC-LUPI, a multi-view LUPI framework with bi-level modality completion. Experimental results demonstrate that this model significantly outperforms existing comparative algorithms and effectively enhances the diagnostic efficacy of medical imaging-based CAD systems utilizing single-modality data buses (48). Han et al. developed a meta-learning-based deep neural network SVM+ algorithm (ML-DSVM+). This model significantly improves classification performance in class-imbalanced scenarios while effectively mitigating overfitting (49).The team developed a novel Dual-Supervised Parameter Transfer Classifier (DSPTC). This algorithm significantly enhances sensitivity and specificity in early breast cancer diagnosis by simultaneously transferring knowledge from both paired data with shared labels and unpaired data with heterogeneous labels, demonstrating substantial translational potential for clinical applications (50). Concurrently, experimental validation on both the ADNI and BBUI datasets confirmed the effectiveness of the dual-supervised transfer classifier (DSTC) developed by Fei et al. (51). With the continuous advancement of artificial intelligence, the intersection of AI and ultrasound holds limitless potential. The path toward precise diagnosis and treatment of breast cancer is bright and promising.

## 5 Restrictions

There are many challenges when empowering the field of breast cancer with artificial intelligence (52). From a technical perspective: (1) Personalizing data collection is difficult; it is challenging to gather information such as genetic data, which is outside of clinical indicators. (2) Data quality needs improvement; there are discrepancies in data among different ethnic groups, and electronic health records are not uniformly structured, leading to a lack of standardized data processing. (3) Code is difficult to share; the reproducibility of AI code is low, and the credibility of diagnostic assistance relies on practical validation to enhance public acceptance.

Regarding the data aspect: (1) Accumulating a certain number of citations for an article takes a significant amount of time. In recent years, high-quality papers have not achieved ideal citation counts, which may likely lead to research bias; (2) Research based on the WOSCC database primarily includes English-language clinical trial archives, which inherently limits the scope of data and introduces a language bias, potentially omitting valuable information from other countries and non-English languages (53). (3) The study exclusively relied on data from the WoSCC database, thereby excluding other potential databases such as Wanfang, Weipu, and PubMed, which may contain valuable studies relevant to the research topic, potentially introducing a selection bias (54, 55). In future studies, we will integrate multiple databases such as CNKI, Scopus, Google Scholar, and PubMed, along with literature from diverse countries and languages, to ensure a more comprehensive and multidimensional analysis of the research topic, actively addressing the limitations and shortcomings of the current study. Although this study has certain limitations, our in-depth discussion overall establishes a solid foundation for understanding research themes, hotspots, and development trends regarding the integration of artificial intelligence with ultrasound technology in breast cancer studies (18, 56).

## 6 Conclusion

This study utilizes CiteSpace and VOSviewer software to conduct a visual analysis of literature on the application of artificial intelligence technology combined with ultrasound in breast cancer research from 2004 to early 2025. It identifies the core authors and collaborative institutions in this field and provides an overarching view of its development. Key findings indicate that core researchers work closely together, forming an academic circle. The strong collaboration within this academic circle plays a crucial role in enhancing the influence of this field (57). The number of articles published by People’s China stands out at the forefront; however, when it comes to influence and the depth of participation, the USA, Germany, and England have the edge. For emerging countries, what is urgently needed at present is to enhance the frequency of collaborative research efforts among nations. From a global perspective, countries should make every effort to establish and maintain close and solid ties with industrialized nations like the USA, in order to promote the advancement of disciplines. Current AI-ultrasound applications in breast oncology demonstrate suboptimal technological maturity, and the overall process is somewhat slow. In the future, the focus in this area will shift, emphasizing the refinement of diagnostic accuracy through deep learning techniques, while leveraging the power of big data to achieve breakthroughs in developing treatment plans and predicting prognosis for breast cancer.

## Data Availability

The original contributions presented in the study are included in the article/supplementary material. Further inquiries can be directed to the corresponding author.
