# Correction: Artificial intelligence in breast ultrasound: a systematic review of research advances

**DOI:** 10.3389/fonc.2026.1823077

**Published:** 2026-03-24

**Authors:** Jiawei Liu, Linping Pian, Jie Chen, Jingjing Zhao, Yameng Liu, Fanbo Meng, Cheng Zeng

**Affiliations:** 1 Henan University of Traditional Chinese Medicine, Zhengzhou, China; 2 The First Affiliated Hospital of Henan University of Traditional Chinese Medicine, Zhengzhou, China; 3 The Third Affiliated Hospital of Henan University of Traditional Chinese Medicine, Zhengzhou, China

**Keywords:** breast cancer, ultrasound, artificial intelligence, VOSviewer, CiteSpace

Affiliation “3 Henan University of Traditional Chinese Medicine, Zhengzhou, China” was omitted for authors Jiawei Liu, Cheng Zeng. This Affiliation has now been added for authors Jiawei Liu, Cheng Zeng.

Author Jiawei Liu, Cheng Zeng was erroneously assigned to Affiliation “1 The First Affiliated Hospital of Henan University of Traditional Chinese Medicine, Zhengzhou, China” and

“2 The Third Affiliated Hospital of Henan University of Traditional Chinese Medicine, Zhengzhou, China”. These Affiliations have now been removed for author Jiawei Liu, Cheng Zeng.

The original version of this article has been updated.

